# Effects of systemic versus local hypoxia on post-activation performance enhancement in well-trained field hockey players

**DOI:** 10.3389/fphys.2026.1708537

**Published:** 2026-05-22

**Authors:** Betul Coskun, William Hampton, Michael J. Hamlin

**Affiliations:** 1 Faculty of Sport Sciences, Erciyes University, Kayseri, Türkiye; 2 Department of Tourism, Sport and Society, Lincoln University, Christchurch, New Zealand

**Keywords:** acute effect, blood flow restriction, normobaric hypoxia, PAPE, plyometrics, recovery

## Abstract

**Background:**

The aim of this study was to investigate the effect of systemic or local hypoxia on the time course of post-activation performance enhancement (PAPE).

**Methods:**

Fourteen well-trained field hockey players (19.1 ± 1.2 years; 75.4 ± 9.3 kg) (n=14; 4 females and 10 males) were randomly given five different priming conditions: HYP (plyometrics in hypoxia), BFR (plyometrics with blood flow restriction), NORM (plyometrics in normoxia), PLC_hyp_ (plyometrics during sham hypoxia) and PLC_bfr_ (plyometrics during sham BFR). The conditioning activity (CA) consisted of 1x5 drop-jumps (DJ), 1x5 countermovement-jumps and, 1x5 single-leg horizontal jumps (on both legs). Hypoxia was conducted under normobaric conditions with SpO_2_ clamped to 87% via a hypoxic generator. BFR was applied with a cuff positioned around the upper thighs with a pressure set to 50% of predicted individual arterial occlusion pressure (AOP). To test the effects of these interventions on PAPE, DJ was measured at baseline and at 3, 6, 9, 12, 15 and 18 min after the CA without the cuffs or mask.

**Results:**

DJ-height (the smallest worthwhile effect, SWE = 0.88cm) and flight-time (SWE = 7.07ms) at 18^th^ min (32.3 ± 5.9 cm; 511.5 ± 46.78 ms) was significantly higher than baseline (30.0 ± 4.4 cm, p= 0.041; 493.1 ± 35.9 ms, p=0.049) after HYP. BFR improved jump height (SWE = 0.93cm) and flight-time (SWE = 7.65ms) at the 3^rd^ min (31.7 ± 5.0 cm; 507.2 ± 39.6 ms) compared to baseline (30.2 ± 4.7 cm, p=0.01; 494.6 ± 38.4 ms, p=0.01) (p<0.05). There was no significant improvement in DJ performance throughout the recovery period for the NORM or placebo conditions (p>0.05).

**Conclusions:**

Jump performance improvement after plyometric CA appeared early with BFR and late with systemic hypoxia. Such large differences in PAPE timing are important aspects to consider when using such PAPE methods.

## Introduction

Post-activation performance enhancement (PAPE) is based on the idea that a maximum or near-maximum muscle contraction (often referred to as the conditioning activity, CA) enables greater performance in voluntary activities in subsequent exercises (Krzysztofik et al., 2022; dos Santos Silva et al., 2023). On the other hand, there is another term, “PAP” (post-activation potentiation) which has been used interchangeably with PAPE (Boullosa et al., 2020). PAP is an augmented muscle contractile response, electrically evoked (twitch force), after an intensive voluntary contraction (Blazevich and Babault, 2019), while PAPE is used for the increased voluntary force production that follows the conditioning exercise (Blazevich and Babault, 2019; Formiglio et al., 2024). After a conditioning activity, changes in the balance between potentiation and fatigue affect the level of subsequent performance and thereby PAP/PAPE. The potentiation resulting from the CA needs to surpass the fatigue level to reveal a performance enhancement (Seitz and Haff, 2016; Doma et al., 2020; Krzysztofik et al., 2022).

Many studies have investigated how manipulating the type, volume, and intensity of the CA affects the PAPE outcome (Krzysztofik et al., 2022), which seems to be most effective in explosive-type actions and performances like sprinting, jumping, and throwing (dos Santos Silva et al., 2023). Current evidence suggests that plyometric type exercises are ideal as conditioning exercises as they fatigue the subject less, compared to traditional loaded resistance exercise (Seitz and Haff, 2016; Sharma et al., 2018) and seem to be associated with the preferential recruitment of fast-twitch motor units, one of the purported mechanisms of PAPE (dos Santos Silva et al., 2023).

Blood flow restriction (BFR) has been suggested to enhance post-activation performance enhancement because the hypoxia arising from BFR induces early fatigue of the slow-twitch muscle fibers, thus triggering the recruitment of fast-twitch fibers (Doma et al., 2020; Tian et al., 2022; Sun and Yang, 2023) thereby enhancing force production. The use of BFR also has the potential to improve stretch-shortening cycle mechanics (Tian et al., 2022) and enhance explosive performance (Wilk et al., 2020). BFR is also commonly used as a CA to remove the potential increased risk of injury associated with traditional high-resistance conditioning exercises (Tian et al., 2022).

With BFR, there is a local effect whereby the exercising muscle downstream of the inflated cuff becomes hypoxic (Girard et al., 2019). However, such exercise can be uncomfortable (Spitz et al., 2022) and even dangerous for people with cardiovascular disease risk (Renzi et al., 2010). An alternative to BFR is to use systemic hypoxia, where the whole body becomes hypoxic and there is no increased vascular pressure due to the cuff inflation. Currently to our knowledge only one study has investigated the use of systemic hypoxia and reported a higher post-activation potentiation after an active re-warm-up under hypoxic conditions (F_I_O_2_ = 0.15) compared to normoxic conditions (Ramos-Campo et al., 2020). Proposed mechanisms behind the improved post-activation potentiation with systemic hypoxia included greater reliance on the anaerobic metabolism, increased blood flow, enhanced oxygen uptake and change in body temperature (Ramos-Campo et al., 2020).

Mechanisms such as intramuscular fluid accumulation, neural change and increased muscle temperature, have been postulated to be involved in producing PAPE (Blazevich and Babault, 2019; Krzysztofik et al., 2022; Sun and Yang, 2023). As for the possible mechanisms for PAP, they have been reported as phosphorylation of myosin regulatory light chains, the recruitment of high threshold motor units, and neural firing rates (Blazevich and Babault, 2019; Sun and Yang, 2023). Moreover, it should not be ignored that PAP may contribute to an increased PAPE response (Blazevich and Babault, 2019; Zimmermann et al., 2020). Therefore, increased PAPE response may be seen after a local or systemic hypoxic CA due to any of the mechanisms mentioned above not only related to PAPE but also related to PAP, in this study.

Another issue that needs to be examined is the time points at which the PAPE response is likely to occur. Doma et al. (2020) found significant jump performance enhancement between the 6^th^ and 15^th^ min, when CA was conducted under BFR conditions. Zheng et al. (2022) reported that PAP can arise earlier and proceed longer with BFR than when conducted under traditional high-intensity protocols. The only research on systemic hypoxia, Ramos-Campo et al. (2020) found performance improvement only at the 3^rd^ min into the recovery period. With such contradictory results it is not clear at what time point enhancement in performance should be expected and therefore when and how a coach might utilise such training in athletes.

There is little consensus as to whether hypoxia, especially systemic, has a beneficial effect on plyometric-driven PAPE. Additionally, the time course of PAPE after hypoxic plyometric exercise is not well researched. Therefore, the aim of this study is to examine the effect of plyometric exercises with and without hypoxia (both systemic and local) as an activation exercise on PAPE, and secondly, to test the time differences in any PAPE response during recovery between five conditions (systemic hypoxia, local hypoxia, normoxia, placebo for systemic hypoxia and placebo for local hypoxia). We hypothesise that normobaric hypoxia (systemic and/or local) given during plyometric conditioning exercise will enhance the normal PAPE response, however we are unsure on the time course of this enhancement.

## Methods

### Study design

**Figure 1:**
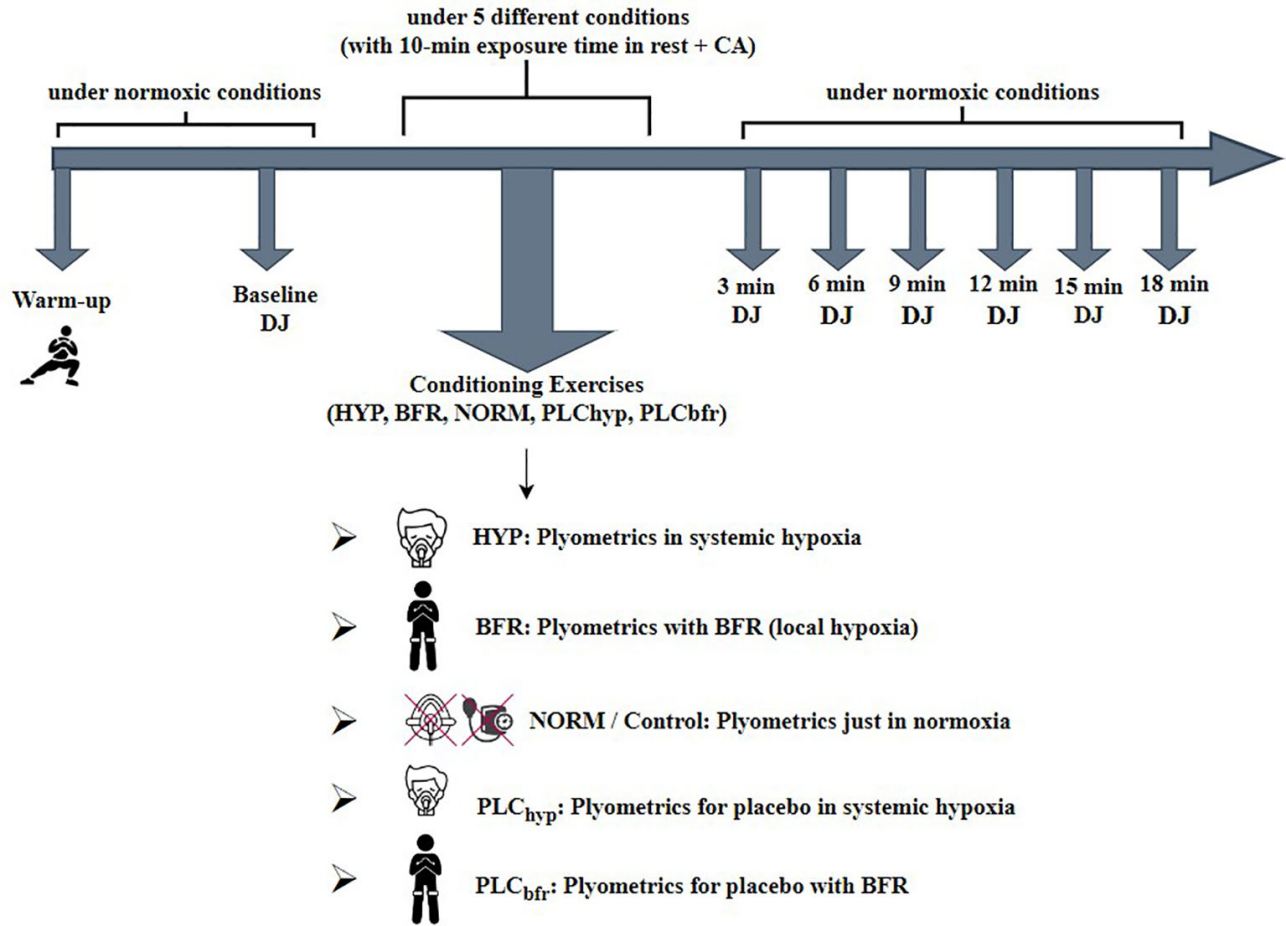
Representation of the experimental design (icons by Leremy, Gravisio, Vector Valley, Milkghost Studio, AomAm, from Flaticon.com).

This study was a randomized crossover study where participants were instructed to cease resistance training 24 hours prior to the first test day and not to resume resistance training until the study was completed. Participants were asked to attend five randomly allocated training sessions each lasting about 1 hour with approximately 24–48 hours between sessions. We used a randomized counterbalanced Latin square design by applying each condition in an unstable order for each participant to control systematic bias arising from cumulative fatigue or a learning effect potentially induced by multiple testing sessions. Also, we compared the baseline jump performances of all five conditions and found no statistically significant differences (p>0.05) and ensured that 24–48 hours between condition sessions was sufficient to prevent any carryover effect from previous condition sessions. Participants were also asked not to consume alcohol and caffeine 24 h prior to any testing session. On each training session, participants completed a drop jump (DJ) test at baseline and following the conditioning activity at 3, 6, 9, 12, 15, and 18 min into recovery (**Figure 1**). The heart rate (HR) and oxygen saturation (SpO_2_) were taken to give an indication of the level of hypoxia and the physiological strain of hypoxia.

Prior to the first official test day a familiarization session was performed which introduced the participants to the types of exercises and the equipment that would be used in the study.

Also, during the familiarisation session, individual drop jump heights were established because individualization is thought to optimize neuromuscular adaptation and its benefits, depending on the individual neuromuscular capacity (Byrne et al., 2010). Participants performed 3 DJs from 5 different jump heights (20, 30, 40, 50, and 60 cm), and the jump height with the greatest RSI (jump height/contact time) from all DJs was considered as individual optimal jump height (Byrne et al., 2010; de Poli et al., 2020). At the end of testing, it was established that the individual optimal jump height was 40 cm for males and 30 cm for females.

### Participants

Fourteen well trained field hockey players with at least 5 years hockey experience, 4 females (age 19.5 ± 1.3 years; body mass 67.7 ± 8.9 kg; body height 167.6 ± 3.2 cm; body fat percentage 23.7 ± 7.2; skeletal muscle mass 28.7 ± 2.0 kg, mean ± SD) and 10 males (age 19.0 ± 1.2 years; body mass 78.5 ± 7.8 kg; body height 181.1 ± 4.4 cm; body fat percentage 12.4 ± 3.3; skeletal muscle mass 38.8 ± 3.0 kg), voluntarily participated in this study (**Table 1**).

**Table 1:** Physical characteristics of the participants.

Variables	Female (n=4)	Male (n=10)	Total (n=14)
Age (years)	19.5 ± 1.3	19.0 ± 1.2	191.1 ± 1.2
Body height (cm)	167.6 ± 3.2	181.1 ± 4.4	177.2 ± 7.4
Body mass (kg)	67.7 ± 8.9	78.5 ± 7.8	75.4 ± 9.3
Body fat percentage (%)	23.7 ± 7.2	12.4 ± 3.3	15.6 ± 6.9
Skeletal muscle mass (kg)	28.7 ± 2.0	38.8 ± 3.0	36.0 ± 5.5
Training experience (years)	11.8 ± 4.7	10.6 ± 2.9	10.9 ± 3.4

For a repeated measures ANOVA (within-between interaction design; 5x7, condition x time points) with an effect size of 0.40 (Doma et al., 2020), 0.80 statistical power, and 0.05 alpha level, we calculated 15 participants for an *a priori* sample size as a result of G*Power analysis (G*Power 3.1.9.7). We started with 16 athletes, but two were excluded from the analyses since they did not participate in all measurement sessions. As a result of the sensitivity analysis for a 5x7 repeated-measures ANOVA, we estimated that a participant number of 14 was sufficiently sensitive for a minimum detectable effect size of 0.23, based on the average correlation among repeated measures (0.83), considering an alpha level of 0.05 and a 0.80 power.

After briefing the participants about the study but keeping the hypothesis confidential, written informed consents were obtained from the participants. The study was approved by Lincoln University Human Ethics Committee (No: HEC2023-12, Date: 17 March 2023) and in accordance with the Declaration of Helsinki.

### PAPE conditions

Initially, a standardized warm-up consisting of 5 min low-intensity cycling (Wattbike Pro, Nottingham, UK) followed by 5 min dynamic stretching of the major lower body muscle groups was undertaken (Chen et al., 2017). Two trials baseline DJ tests were performed 1 min after warm-up. Participants were asked to jump as high as possible with an effort of a minimum ground contact time during DJs (**Figure 1**).

Participants were then given a 10-min exposure to the particular condition at rest (e.g. the hypoxic masks and blood pressure cuffs were attached to the participants for 10 min while resting before the conditioning activity). Through this exposure time, HR and SpO_2_ were measured to determine the overall hypoxia effect, and to see the difference in HR and S_P_O_2_ during rest and exercise between conditions. The mean values of the last 5 min at rest were used to evaluate resting HR and S_P_O_2_ under each particular condition. The plyometric exercises were then performed under the PAPE condition protocol and then cuffs and masks were removed, and DJ tests were completed in normoxia for all conditions during the 18 min of recovery (**Figure 1**).

Based on previous studies (Tobin and Delahunt, 2014; Chen et al., 2017; Doma et al., 2020; Ramos-Campo et al., 2020; Munshi et al., 2022; Zheng et al., 2022) conducted on trained athletes similar to those in this study and on information from the coach, we used a PAPE conditioning exercise with only 5 reps on 3 different exercises (DJ, CMJ, single-leg horizontal jump for both legs). Repetitions were applied with 15-sec rest between each rep and 2-min rest between different exercises. After completing each exercise of CA, and at rest between exercises, participants’ HR and SpO_2_ were measured.

In addition to the widespread use of DJ protocols as CA in such studies, it was reported that a DJ protocol of a study could generate both PAP and PAPE as a result of the same research (de Poli et al., 2020; Moura Zagatto et al., 2022). On the other hand, the type of activity/exercise following the CA has the potential to affect the PAP/PAPE outcome. It is known that high similarity between the CA and the subsequent exercise is likely to lead to high potentiation (Formiglio et al., 2024). Therefore, the DJ test was used to examine the PAPE effect.

Although it is not possible to equalize the level of hypoxia for local and systemic hypoxic protocols, the hypoxic levels recommended in the literature have been used for both, (a moderate level of AOP for blood flow restriction) (Loenneke et al., 2015b; Zheng et al., 2022) and an SpO_2_ of 87% for hypoxia (HYP) (Ramos-Campo et al., 2020). The normoxic condition (NORM) was performed in normobaric normoxia without using any hypoxic device/pressure cuff. For the placebo conditioning, the participants used the same equipment (either a face mask attached to the hypoxicator (PLC_hyp_) or blood pressure cuffs attached to the upper thigh (PLC_bfr_), but the face mask delivered normoxic room air and the cuff pressures were set very low (15-20% of predicted AOP).

### Protocol for HYP condition

HYP was performed under normobaric conditions via a face mask attached to a hypoxic generator (GO2Altitude hypoxicator, Biomedtech, Victoria, Australia). The hypoxicator automatically adjusted the fractional inspired concentration of oxygen (F_I_O_2_) through a biofeedback control system to maintain a moderate level of hypoxia (SpO_2_ of 87%).

In the study conducted in real altitude, changes were found in DJ height in favor of higher altitude (2.320 m) compared with low altitude (690 m) (Štirn et al., 2022). However, Scott et al. (2017) in their study in which they investigated acute responses to moderately loaded resistance exercise in hypoxia, stated that no significant hypoxia dose was observed at the muscle level and this may be due to the use of moderate hypoxia (F_I_O_2_ = 16%). Contrary to this, Solsona et al. (2021) found increased local hypoxia within the working muscles resulting from sprint interval exercises under hypoxic conditions at 13% F_I_O_2_. On the other side, in the study of Coşkun et al. (2022), which tested the effects of a pure plyometric training applied under normobaric hypoxic conditions, a level of normobaric hypoxia equal to nearly 3536 m (F_I_O_2_ of 0.135) was used and a greater increase was observed in some jump performances (drop jump and squat jump) compared to the training under normoxic conditions. Although there is no study aiming to investigate the PAP effect of a plyometric CA in systemic hypoxia, Ramos-Campo et al. (2020) found a PAP effect after a re-warm-up activity consisting of horizontal jumps applied at F_I_O_2_ = 15% hypoxia (mean of SaO_2_ (%) = 87.90) in their study.

### Protocol for BFR condition

In the BFR conditioning protocol, plyometric exercises were performed with pressure cuffs (10 cm width, Sports Rehab Tourniquet, SportsRehab, Australia) attached to the upper-most thigh area with the cuff pressure set to 50% of predicted individual AOP. We estimated individual maximum occlusion pressures by measuring the lower body blood pressure (measured in the posterior tibial artery at the ankle using the asculatory method), thigh length, and thigh circumference (Seca metal tape) which were inputted into the BFR pressure calculation tool provided by the cuff manufacturer (Sports Rehab Tourniquet, SportsRehab, Australia), which was developed based on previous research (Loenneke et al., 2015a). Lower-limb occlusion pressure was determined using the regression equation reported by Loenneke et al., (2015a), including these measurements of thigh circumference and systolic and diastolic blood pressure, since the thigh circumference was reported as the primary determinant of AOP for the lower body (Loenneke et al., 2015a). For cuff pressure, we used 50% of the estimated limb occlusion pressure, agreeing with recommendations to prescribe BFR relative to the individual limb occlusion pressure (Loenneke et al., 2015b; Patterson et al., 2019).

The estimated individualized AOP of the participants was determined to be 147.9 ± 18.5 mmHg (ranging from 120 to 195 mmHg). The relative pressure mean was 73.9 ± 9.2 mmHg (ranging from 60 to 97.5 mmHg) according to 50% of individual AOP for the BFR condition, and it was 25.1 ± 2.0 mmHg (ranging from 20.0 to 29.0 mmHg) according to 15-20% of individual AOP for the PLC_bfr_ condition.

Regarding safety, we used an individualized cuff pressure rather than applying a constant pressure for all and also preferred a recommended percentage (50%) of AOP. We considered individual anthropometric differences (like limb circumference) and provided safe cuff pressures as recommended in the literature. Also, we constantly observed participants physiologically for subjective tolerance by measuring heart rate and RPE taken immediately after BFR-CA exercises and ensured that participants stayed within safe limits for cardiovascular strain. We observed that all participants tolerated the prescribed pressure without adverse effects. Exercises were performed with the application of continuous BFR since the continuous BFR has a greater effect during exercise, compared to intermittent BFR (Zheng et al., 2022).

### Drop jump test

The DJ was performed with hands on hips either from a 40 cm height (males) or 30 cm (females). Participants were asked to jump twice (with a 30-sec rest between jumps) for the baseline measurement (mean of two jumps were used for the analysis and also intraclass correlation coefficient (ICC) was calculated for two trials of the baseline), and once at each of the time points after completing the conditioning exercises. The criterion for the jump to be considered valid was dropping (not jumping) from the box to the jump mat (SmartJump Portable Jump Mat, Vald Performance Pty Ltd, Brisbane, Australia) with no hip/knee flexion during the flight phase and then jumping as high as possible with as little time on the ground as possible (Yang et al., 2022). Immediately after each DJ test (within the first 15 seconds), participants’ HR, SpO_2_, and RPE (Borg’s 6–20 scale) were recorded. Also at rest between the jump tests we continued to record the HR and SpO_2_.

The performance variables of the DJ test, contact time (CT), flight time (FT), jump height, and reactive strength index (RSI) were evaluated for the analyses.

### Statistical analysis

Descriptive data were expressed as the mean ± SD. After examining the normal distribution of data with Shapiro–Wilk test, a 5x7 (condition x time) repeated measures analysis of variance (Condition: HYP, BFR, NORM, PLC_bfr_, PLC_hyp_) (time: at baseline, 3^rd^, 6^th^, 9^th^, 12^th^, 15^th^, 18^th^ min of recovery) was used to investigate any differences between conditions and time. To check the assumption of variance, Mauchly’s test of sphericity was used and the Greenhouse–Geisser correction was applied for sphericity violation. When there was a significant main effect or interaction, we applied follow-up analyses. Especially for significant interactions, we examined simple main effects to detect the differences between conditions at each time point. We applied the Bonferroni adjustment for all pairwise comparisons. Significance level was considered as <0.05. All statistical analyses were completed using the Statistical Package for Social Sciences (version 28) (SPSS Inc. Chicago, IL, USA). Partial Eta Squared (partial η^2^) was used as an estimate of the effect size. The smallest worthwhile effect (SWE) for DJ height and FT was computed for each condition with the standard deviation (SD) of all baseline DJ height and FT multiplied by 0.2 (Lum and Chen, 2020).

## Results

**Figure 2:**
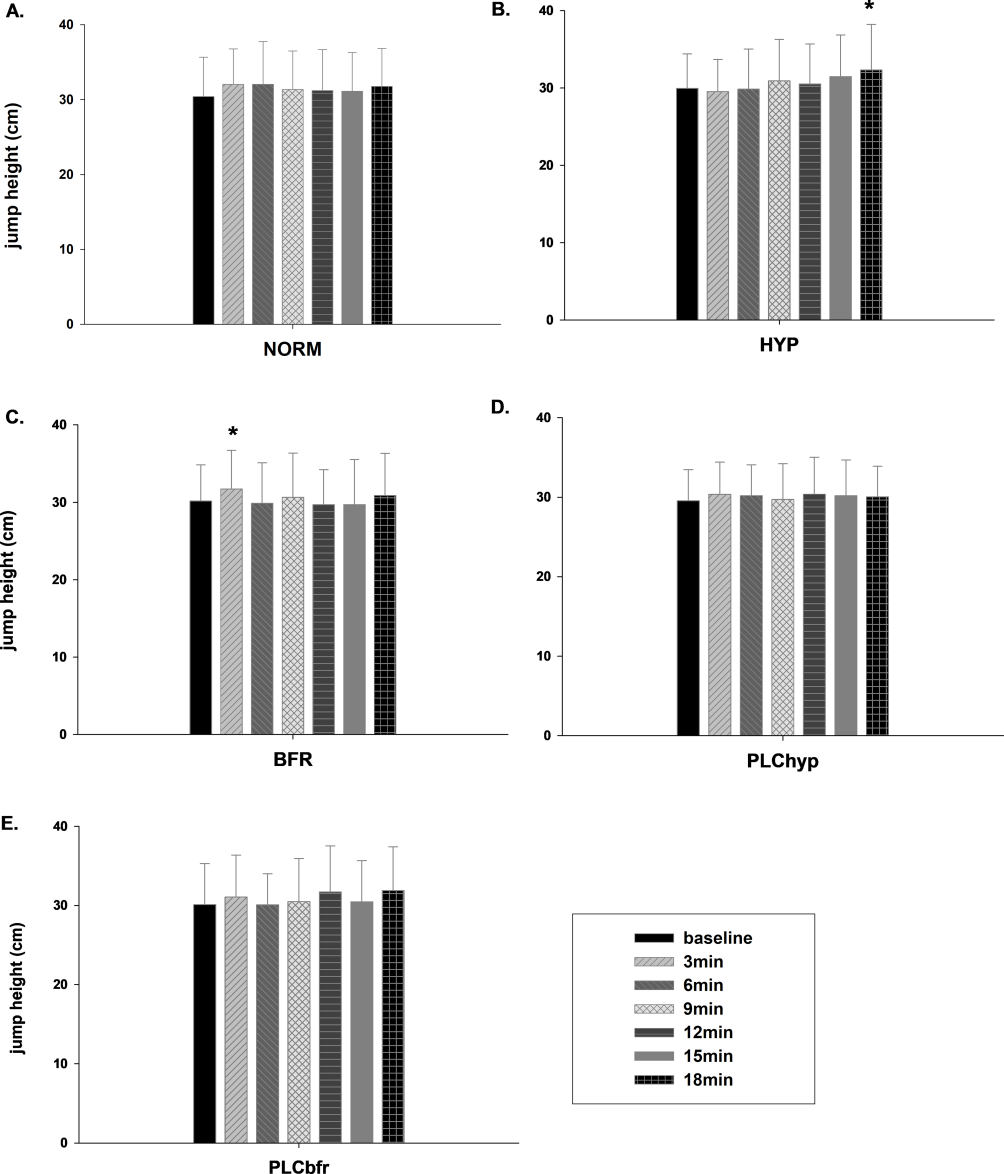
The mean ± standard deviation at baseline, 3, 6, 9, 12, 15 and 18 min after jump exercises for the jump height of DJ test for five different conditions, NORM **(A)**, HYP **(B)**, BFR **(C)**, PLChyp **(D)**, PLCbfr **(E)**. * Significantly higher than baseline of the same condition (p<0.05).

As a result of the two-way repeated measures ANOVA, for DJ height, the sphericity assumption was met for the main effect of condition, but violated for time. While there were no significant main effects of condition (sphericity-assumed values based on Mauchly’s Test; F (4, 52)= 1.34, p=0.268, partial η^2^ = 0.09), there was a significant main effect of time (corrected values based on Greenhouse-Geisser; F(3.03, 39.37)= 3.7, p= 0.020, partial η^2^ = 0.22) on participants’ DJ height. Also, a significant interaction was found between condition and time (Greenhouse-Geisser; F(7.28, 94.64)= 2.4, p= 0.025, partial η^2^ = 0.16). According to pairwise comparisons, DJ height at the 18^th^ min (32.3 ± 5.9 cm) was significantly higher than baseline (30.0 ± 4.4 cm, p= 0.041) after HYP and was greater than the smallest worthwhile effect (0.88 cm). The reliability of the baseline jump performance was an ICC of 0.94 (95% CI: 0.26-0.99) based on average measures. On the other hand, compared to baseline (30.2 ± 4.7 cm) a higher jump height at the 3^rd^ min (31.7 ± 5.0 cm, p= 0.01) was found in the BFR condition (**Figure 2**) which was also higher than the smallest worthwhile effect of 0.93 cm. The reliability of the baseline jump performance was an ICC of 0.96 (95% CI: 0.03-0.99) based on average measures. There was no significant improvement in DJ performance throughout the recovery period for the NORM or placebo conditions (p>0.05).

There was no significant main effect of condition (sphericity-assumed values based on Mauchly’s Test; F(4, 52)= 1.2, p= 0.32, partial η^2^ = 0.09), however there was a significant main effect of time (corrected values based on Greenhouse-Geisser; F(3.13, 40.71)= 3.7, p= 0.017, partial η^2^ = 0.22) on participants’ FT, and a significant interaction between condition and time (based on Greenhouse-Geisser; F(7.35, 95.60)= 2.3, p= 0.028, partial η^2^ = 0.15). Similar to DJ height, pairwise comparisons showed that FT at 18^th^ min (511.5 ± 46.78 ms) showed a worthwhile improvement compared to baseline (493.1 ± 35.9 ms, p= 0.049), after HYP (SWE = 7.07ms) and higher and worthwhile improvement at the 3^rd^ min (507.2 ± 39.6 ms) compared to baseline (494.6 ± 38.4 ms, p= 0.010) after the BFR (SWE = 7.65ms). The reliability of the baseline FT performance was found to be an ICC of 0.93 (95% CI: 0.24-0.98) for HYP and an ICC of 0.96 (95% CI: 0.03-0.99) for BFR, based on average measures.

**Figure 3:**
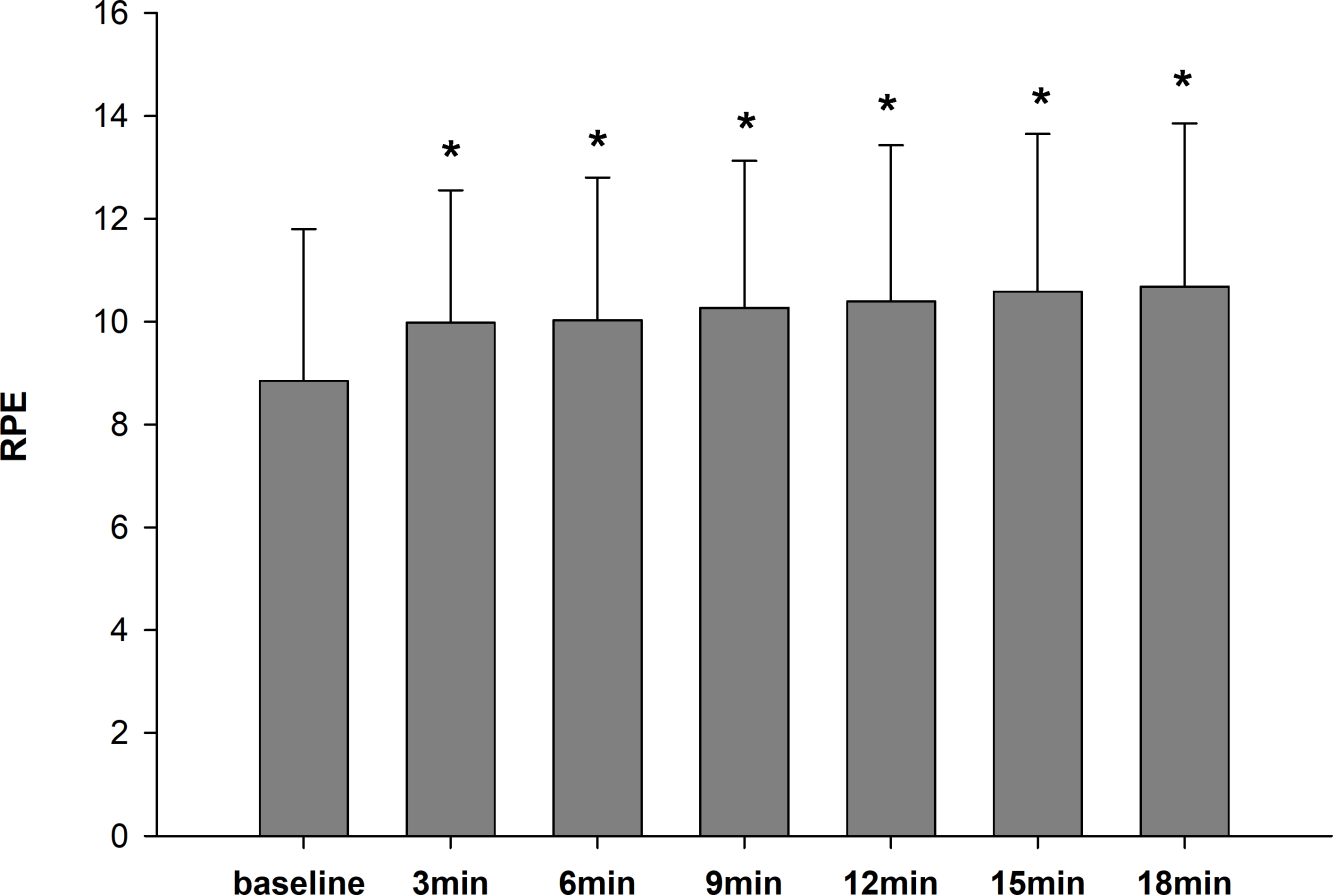
The RPE mean ± standard deviation at baseline, and 3, 6, 9, 12, 15and 18 min after CA. * Significantly greater than RPE at baseline (p<0.05).

**Figure 3** presents the differences of RPE between the time points. There was no condition difference (Greenhouse-Geisser; F(2.66, 34.56)= 3.0, p= 0.051, partial η^2^ = 0.19.) or no significant interaction (Greenhouse-Geisser; F(6.91, 89.81)= 1.2, p= 0.31, partial η^2^ = 0.08). We observed a significant main effect only for time (Greenhouse-Geisser; F(1.69, 22.02)= 12.4, p<0.001, partial η^2^ = 0.49). RPE was significantly lower at baseline (8.9 ± 3.0) than at each subsequent time point (p<0.05).

There was no significant main effect of condition (Mauchly’s Test; F(4, 52)= 1.7, p=0.16, partial η^2^ = 0.12) and time (Mauchly’s Test; F(6, 78)= 1.3, p= 0.25, partial η^2^ = 0.09) or interaction (Mauchly’s Test; F(24, 312)= 1.3, p= 0.19, partial η^2^ = 0.09) for contact time (CT) of the DJ test. No significant main effect of condition (Mauchly’s Test; F(4, 52)= 1.3, p= 0.29, partial η^2^ = 0.09) and time (Greenhouse-Geisser; F(3.45, 44.81)= 0.21, p= 0.91, partial η^2^ = 0.02) or interaction (Greenhouse-Geisser; F(7.86, 102.16)= 1.7, p= 0.11, partial η^2^ = 0.12) was found for RSI(reactive strength index).

There was no significant main effect of condition (Mauchly’s Test; F(4, 52)= 0.7, p= 0.61, partial η^2^ = 0.05) and time (Mauchly’s Test; F(6, 78)= 1.8, p= 0.11, partial η^2^ = 0.12) or interaction (Mauchly’s Test; F(24, 312)= 1.1, p= 0.29, partial η^2^ = 0.08) for HR of the DJ test. Significant main effect of condition (Greenhouse-Geisser; F(2.01, 26.13)= 4.6, p= 0.019, partial η^2^ = 0.26) and time (Greenhouse-Geisser; F(2.34, 30.46)= 6.3, p= 0.003, partial η^2^ = 0.33) was found for SpO_2_ of the DJ test, but after applying Bonferroni correction forpairwise comparisons there was no significant difference (p>0.05). Also, no significant interaction (Greenhouse-Geisser; F(2.77, 36.04)= 2.6, p= 0.07, partial η^2^ = 0.17) was found for SpO_2_ of the DJ test.

**Figure 4:**
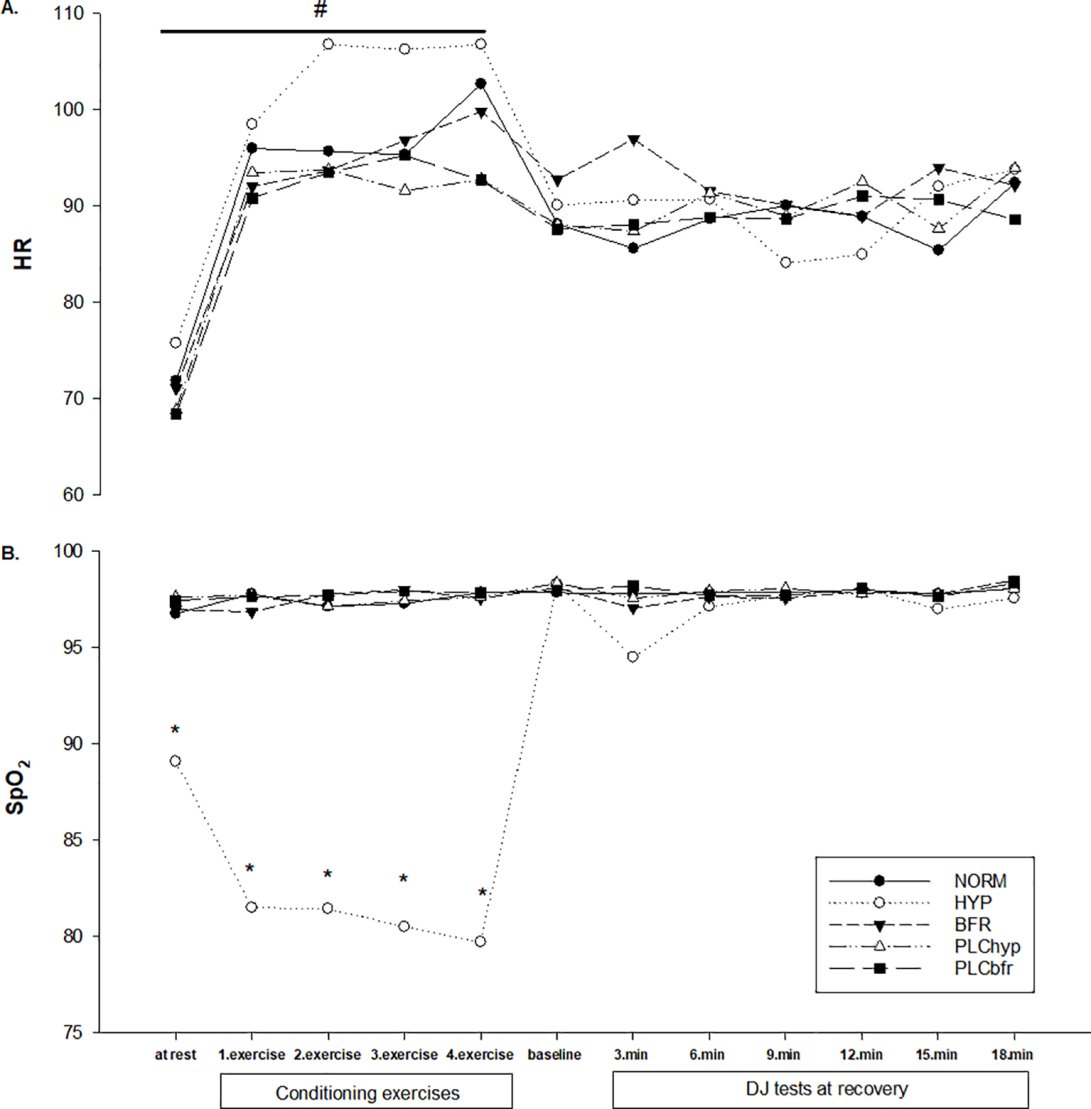
Changes in heart rate **(A)** and S_P_O_2_**(B)** between conditions during conditioning exercises and DJ tests. * Significantly lower than the other conditions (p<0.05). ^#^ Significantly higher in HYP condition than the other conditions (p<0.05).

However, in HR during rest and conditioning exercises, there was a significant main effect for both condition (Mauchly’s Test; F(4, 52)= 8.4, p<0.001, partial η^2^ = 0.39) and time (Mauchly’s Test; F(4, 52)= 100.9, p<0.001, partial η^2^ = 0.89). There was no significant interaction (Mauchly’s Test; F(16, 208)= 1.3, p= 0.18, partial η^2^ = 0.09). The HYP condition had significantly higher HR values compared to the other conditions (**Figure 4A**) (p<0.05). For SpO_2_ during rest and conditioning exercise, there was a significant main effect for condition (Greenhouse-Geisser; F(1.11, 14.38)= 183.2, p<0.001, partial η^2^ = 0.93), for time (Greenhouse-Geisser; F(2.22, 28.82)= 6.2, p=0.005, partial η^2^ = 0.32) and a significant interaction between condition and time (Greenhouse-Geisser; F(3.00, 39.01)= 11.9, p<0.001, partial η^2^ = 0.48). SpO_2_ results for HYP were significantly lower compared to all other conditions (**Figure 4B**) (p<0.05).

**Figure 5:**
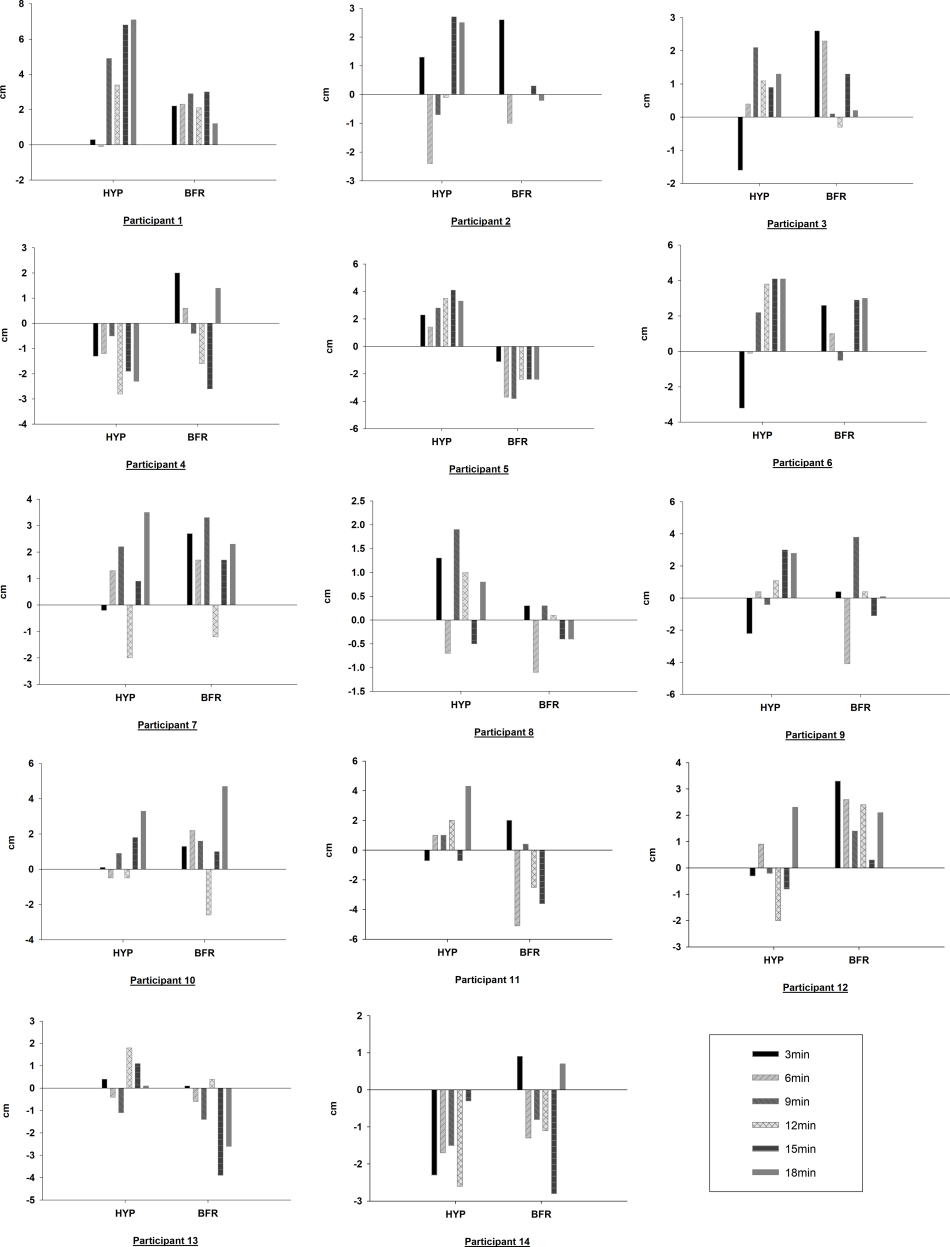
Change (cm) from baseline in DJ height for each participant for HYP and BFR conditions.

To get an indication of the variability of the PAPE response, individual responses to the DJ tests after CA in the HYP and BFR conditions are given in **Figure 5**. Responses with HYP condition ranged from an improvement in DJ performance of 24% (at 18^th^ min) for participant 1 to a drop in performance of 10.8% (12 min) for participant 4. The BFR condition showed a smaller variability with participant 10 showing an improvement of 14.3% (18 min) to -14.1% (6 min) for participant 11. Based on the jump performance enhancement at the 18^th^-minute measurement, which was statistically significant for HYP, comparing the individual responses with the SWE threshold (0.88 cm), 64.3% of participants (n=9) were found to be positive responders, 21.4% (n=3) were non-responders, and 14.3% (n=2) were negative responders. As for the jump performance enhancement at the 3^rd^ minute measurement, which was found to be significant for the BFR, considering the individual responses according to the SWE (0.93 cm), 42.9% of participants (n=6) were positive responders, 50.0% (n=7) were non-responders, and only one participant (7.1%) was a negative responder.

## Discussion

The aim of the present study was to examine the PAPE responses induced by jump exercises with (both systemic and local) and without hypoxia and also to reveal the time differences in any PAPE response during recovery between different conditions. To the best of our knowledge, this is the first study to compare the effects of normobaric normoxia and systemic and local hypoxic conditions on the PAPE response. Our results indicated that PAPE is unlikely to occur without an additional stimulus (local or systemic hypoxia) as we found no significant PAPE effect during the normoxia or placebo conditions. While there was a nonsignificant increase in jump performance at 3^rd^ min and 18^th^ min of NORM, the improvement at 18^th^ min was significant and worthwhile when combining systemic hypoxia with CA. Similarly, combining local hypoxia with the CA resulted in worthwhile and statistically significant improvements in DJ performance at 3 min post CA. The interesting result of our study is the time difference in the PAPE effect between BFR and HYP interventions.

Current theory suggests that after a CA there is an equilibrium period where fatigue or potentiation potentially exists. If potentiation is dominant during this period, there is a subsequent increase in muscular performance, but if fatigue is dominant, reduced performance ensues (Seitz and Haff, 2016; Sharma et al., 2018; Krzysztofik et al., 2022). Our study has shown that the equilibrium between fatigue and potentiation is different for individual players and even different within the same players over an 18 min recovery time.

Literature supports the idea that the PAPE/PAP effect can be seen up to 20 min following a CA (Chiu et al., 2003; Miller et al., 2018; Krzysztofik et al., 2022). Miller et al. (2018) added BFR to whole-body vibration to test the PAP effect. These authors chose a 10-min rest after CA due to previous studies suggesting that peak PAP can be seen between 8 and 12 minutes, but the researchers did not find further PAP response with the addition of BFR (Miller et al., 2018). On the other hand, in the study of Zheng et al. (2022), while PAP seems to occur earlier and lasts longer with BFR procedures (immediately after the activation, 4 min and 8 min after), it is found later in the traditional high-intensity procedure without BFR (8 min after). In the only study to use systemic hypoxia, Ramos-Campo et al. (2020) found CMJ and sprint performance improvement at the 3^rd^ min into the recovery period after jump exercise as re-warm-up activity and concluded that post-activation potentiation may be higher after an active re-warm-up when applied under hypoxia than under normoxic conditions. Doma et al. (2020) found that lunge exercises with BFR significantly improved jump height (~4.5%), and flight time (~3.4%) within 6–15 min after CA. We found nearly the same amount of improvement (5.2% for jump height, 2.6% for flight time) but earlier, at the 3^rd^ min when combined with BFR. However, when CA was combined with HYP we found higher (7.9% for jump height, 3.7% for flight time) but delayed performance improvement (at 18^th^ min). Importantly, both of these performance potentiations were clinically worthwhile and would under normal circumstances result in meaningful improvements in performance for the participants.

An early performance enhancement found with BFR in our study, made us think that a possible PAP response might have contributed to an increased PAPE response (Blazevich and Babault, 2019; Zimmermann et al., 2020). PAP and PAPE have different time points of occurrence. PAP is stated to occur in a shorter time, whereas PAPE is seen in relatively longer periods (Boullosa et al., 2020). It is known that PAP is significant for short periods such as a few minutes (usually less than 3 minutes), while the highest voluntary performance increase usually appears 6–10 minutes after CA (Blazevich and Babault, 2019). However, it is supported by the literature that PAPE improves when PAP has excessively high values (Zimmermann et al., 2020). A contribution of a PAP response may be possible for our PAPE response induced by BFR because the mechanisms related to motor unit recruitment and firing frequency which are similar to the mechanisms proposed for the explanation of PAP (Hamada et al., 2003; Pearson and Hussain, 2014; Miller et al., 2018; Blazevich and Babault, 2019; Sun and Yang, 2023). It is commonly found that with BFR the exercising tissue distal to the inflated cuff becomes hypoxic (Scott et al., 2014; Doma et al., 2020; Aghaei et al., 2023), which causes an earlier recruitment of Type II muscle fibers (as the firing of Type I fibers decrease due to the hypoxic state). Previous research has shown a reduction in the thresholds of these motor units, along with increased firing rates after BFR (Fatela et al., 2019).

On the other hand, it is known that both systemic hypoxia (Ramos-Campo et al., 2020) and BFR (Scott et al., 2014; Doma et al., 2020) might lead to higher type II muscle fibre recruitment and more reliance on anaerobic metabolism which could possibly play a role in the outcome of this study. And also, changes in muscle or cellular water content, which is partly the explanation for PAPE, can improve muscle force generation and this development may be higher in type II fibers (Blazevich and Babault, 2019). Exercise in hypoxia causes compensatory vasodilation (Scott et al., 2014; Aebi et al., 2019; Ramos-Campo et al., 2020), but BFR- and systemic hypoxia-induced vasodilation mechanisms differ from each other (Willis et al., 2019) which may explain the PAPE timing differences found in this study. Previous research has shown that blood flow increases significantly in the early periods after cuff removal (Mouser et al., 2017) which may result in quicker force recovery under the BFR conditions found in this study (Husmann et al., 2018).

It seems that the HYP condition made the exercise more intense. The load of the CA, of which results are also seen under the normoxic condition, might not be sufficient to reveal a potentiation effect, but the additional stress caused by the low O_2_ existence could have led to an increase in the stress of the exercise, inducing some additional effects on the following exercise (Ramos-Campo et al., 2020). We found significantly higher HR during conditioning exercises only under HYP compared to other conditions. Exercise in hypoxia requires an increase in cardiac output to supply sufficient oxygen to the working muscles (Rosales et al., 2022). Also, it is known that the time interval between CA and the subsequent testing exercise is important for PAP/PAPE and high intensities require longer times as recovery for optimum improvement (Formiglio et al., 2024). We found the significant PAPE effect after a longer recovery time only with the HYP condition which may suggest a longer recovery is required when using systemic hypoxia.

Although clarifying the physiological differences were not the main purpose of this study, we speculate the motor unit recruitment as a possible reason for our results since plyometric exercises seem to be associated with the preferential recruitment of type II motor units, one of the mechanisms supporting post-activation potentiation (Seitz and Haff, 2016; Sharma et al., 2018). Furthermore, the mechanism behind the BFR (Doma et al., 2020) and HYP (Ramos-Campo et al., 2020) is also reported to be related to fast-twitch muscle fibre recruitment, and it is known that higher post-activation potentiation appears to be related to fast type II muscle fibers (Hamada et al., 2003). Since fiber type is considered the primary muscle feature which impacts the relationship between potentiation and fatigue (Hamada et al., 2003), and our results showed high inter-individual variability, the muscle fibre type difference may be the potential reason for both conditions’ differences. Fast-twitch type II fibers reveal higher PAP effects, but they are more sensitive to fatigue compared to slow-twitch type I fibers (Hamada et al., 2003). Although both (HYP and BFR) were supported by literature in favour of Type II muscle fibres, the difference in the PAPE effect timing made us think that there may be some differences related to muscle fibre type/subtype. Another possible reason for post-activation potentiation caused by a CA with systemic hypoxia was suggested as body temperature in the study of Ramos-Campo et al. (2020). However, it is seen in the 3^rd^ min in their research, while PAPE effect was seen 18^th^ min in our study. We did not measure muscle temperature, but even if we knew the muscle temperature, we could not still explain the muscle type differences by the muscle temperature increase because all muscle types are prone to display similar dependency on temperature (Blazevich and Babault, 2019). We would have to conduct further research to investigate the effects of muscle fibre composition and also muscle temperature on PAPE outcomes.

The literature emphasizes that the PAPE response is very subjective and shows noteworthy differences among individuals, so individuality should be taken into consideration when athletes may be considering this type of training (Formiglio et al., 2024). The individual changes in jump performance we found within the whole time period when combining CA with HYP varied between a decrease of 10.8% (at 12^th^ min) with participant 4, to an improvement of 24% (at 18^th^ min) with the participant 1. Similarly, variability when using BFR ranged between a decrease of 14.1% (at 6^th^ min) with participant 11, and an improvement of 14.3% (18^th^ min) with participant 10. While only participant 5 responded negatively, participants 1 and 12 responded positively for all time points in the BFR condition indicating considerable variation within and between participants. Our results confirm the large individual differences in response to a conditioning activity. As recommended by the previous studies, evaluations should be individual (Till and Cooke, 2009; Lim and Kong, 2013). We suggest coaches and athletes need to trial such training before any decision to use this type of training occurs.

The PAP/PAPE result is greatly related to the factors of exercise prescription such as the exercise used as CA, volume of CA, and intensity of exercise (Formiglio et al., 2024). We applied the same CA exercises with the same number of sets and repetitions and the same rest intervals for all conditions. However, we found a different timing effect in the PAPE response between BFR and HYP. We showed that with systemic hypoxia, jump performance improvement did not significantly increase until the 18^th^ min into recovery, whereas for the BFR condition, the PAPE effect was much earlier (3^rd^ min). While an early PAPE may be hypothesized to be caused by a significant PAP response, the reason for the delayed PAPE response is reported as the coexistence of potentiation and fatigue. It is not possible to generalize an optimal rest period because recovery durations indicate high inter-individual variability, which is related to various factors such as training experience and strength level (Blazevich and Babault, 2019). Also, as it is known, when CA (conditioning activity) and subsequent exercise are highly similar, the potentiation can also occur to a high degree (Formiglio et al., 2024). Although DJ, CMJ, and single-leg horizontal jumps were performed in our protocol as CA, and DJ was used as the performance test to provide a maximized potentiation effect, the transfer of these effects to a real-game performance still needs to be explored. Therefore, for future studies, it is recommended to design game-simulated test protocols to confirm the effects of these PAPE protocols and to find out whether there are any practical advantages in the unpredictable competitive game circumstances. We have limitations to explain the possible physiological reasons for our results. The important limitation of this study is the lack of muscle oxygenation (e.g., measured by near-infrared spectroscopy), blood lactate, muscle activation (e.g., measured by surface electromyography), muscle temperature, or PAP measurement, which are the most related and common factors associated with postulated mechanisms of PAPE. Therefore, the explanations regarding potential physiological mechanisms discussed in this study should be considered hypotheses rather than precise findings. Although our testing protocol was designed to minimize fatigue and warmup effects in order to see a real performance enhancement, taking single jump measurements for each time point after CA is also a limitation of this study. However, we intentionally conducted a single jump measurement to prevent a potential cumulative fatigue induced by multiple maximal exertions at seven testing time points. To confirm that this single trial did not risk the reliability of our findings, we also examined ICC based on the single measures as well. For instance, the reliability of baseline jump height was found to be an ICC of 0.88 (95% CI: 0.15–0.97) for HYP and 0.93 (95% CI: 0.02–0.99) for BFR based on the single measures. These results demonstrate that a single-trial measurement may reveal a representative performance result and may diminish the risk of Type II error. Furthermore, although we randomized the trial order to minimize systematic bias, we could not control the menstrual cycle periods of the female athletes, which should be taken into consideration when discussing the results. Though the study has the power of the crossover design, the small sample size is still a limitation of the study. Although our G*Power analysis showed that this sample size was acceptable based on medium to large effect sizes, for future studies, larger sample sizes estimated based on the small effect sizes are also recommended to improve the generalizability. Furthermore, whether there would be gender-specific differences in performance or physiological outcomes is a necessary research scope for future studies. In future studies, the common effects of BFR and HYP, such as neural activity and vasodilation, as well as the presence of a potential PAP effect for BFR-induced PAPE results and increased muscle temperature for HYP-induced PAPE results should be examined.

Though we did not find a large PAPE effect, the possible benefits of even small performance improvements for competitive athletes should not be undervalued. After further research, plyometric CAs combined with systemic or local hypoxia may be recommended according to the needs of athletes. For instance, BFR may be recommended for training/matches requiring early performance increase, whereas systemic hypoxia (HYP) may be used when performance enhancement does not require immediate improvement.

## Conclusion

While the same plyometric conditioning protocol in normoxic or placebo conditions had no significant PAPE effect, we found an early jump performance improvement after a conditioning activity that involved BFR and a later jump performance improvement when using systemic hypoxia in conjunction with the conditioning activity. Because of the large variability of the individual responses to PAPE, we suggest that PAPE should be considered individually for each athlete.

## Data Availability

The raw data supporting the conclusions of this article will be made available by the authors, without undue reservation.
